# Artificial intelligence model as a tool to predict prediabetes

**DOI:** 10.1038/s41598-025-23227-0

**Published:** 2025-12-08

**Authors:** Aarthi Yesupatham, Raja Das, Go Bharani, Meera Shaikmeeran, Radha Saraswathy

**Affiliations:** 1 Department of Biomedical Sciences, 120TT Biomedical Genetics Research Laboratory, School of Biosciences and Technology, Vellore Institute of Technology, Vellore, Tamil Nadu India; 2 Department of Mathematics, School of Advanced Sciences, Vellore Institute of Technology, Vellore, Tamil Nadu India; 3 Department of General Medicine, Mothers Care Diabetes Centre, Vellore, Tamil Nadu India; 4 Department of Community Medicine, Government Vellore Medical College, Vellore, Tamil Nadu India

**Keywords:** Prediabetes, Biochemical parameters, Artificial intelligence, Pattern neural network, Support vector machines, K-Nearest neighbors (KNN), Logistic regression, Biomarkers, Computational biology and bioinformatics, Diseases, Endocrinology, Medical research

## Abstract

Prediabetes is characterized by elevated blood glucose levels that are higher than normal but below the threshold for diabetes mellitus. While AI models have been used for prediabetes prediction, most rely solely on standard clinical and biochemical markers. This study introduces a novel Pattern Neural Network (PNN) model that uniquely integrates total antioxidant scavenging potential with traditional risk factors, providing new insights into the role of oxidative stress in prediabetes risk stratification among Indian adults. A total of 199 individuals aged 18 to 60 years were recruited and classified based on HbA1c levels into Control (*n* = 99) and Prediabetes (*n* = 100) groups. Fourteen input features including age, gender, total antioxidant status, HbA1c, FBG, OGTT, TGL, HDL, LDL, VLDL, TC, WC, Hb, and BMI were used to train a PNN with 14 input nodes, 10 hidden nodes, and one output node. The dataset was randomly divided into training, validation, and testing subsets. Model performance was compared against SVM, KNN, and LR classifiers. Feature importance analysis was conducted to interpret the model’s clinical relevance. The PNN model achieved superior validation performance with an accuracy of 98.3%, outperforming SVM (96%), KNN (83%), and LR (71%). Notably, antioxidant scavenging potential and waist circumference emerged as the most influential predictors. The model’s output value of 0.8770 (threshold > 0.5) effectively identified individuals at increased risk of developing diabetes. By incorporating oxidative stress markers, this study provides the first AI model in an Indian cohort to link antioxidant status to prediabetes risk. The PNN model demonstrates excellent accuracy and interpretability, offering a clinically actionable tool for early disease detection and personalized intervention.

## Introduction

Prediabetes is a significant risk factor for developing diabetes, particularly in individuals with Impaired Fasting Glucose (IFG) and Impaired Glucose Tolerance (IGT)^1^. Studies indicate that approximately 5–10% of people with prediabetes progress to diabetes each year, while a similar percentage revert to a healthy state^2^. In today’s healthcare landscape, early detection and management of chronic conditions like prediabetes are essential to prevent the development of more severe complications, such as type 2 diabetes.

Traditional methods for diagnosing prediabetes typically rely on a combination of clinical tests and subjective assessments, which may take longtime, expensive and may not accurately predict individual risk. Therefore, artificial intelligence (AI) models have emerged as promising tools for early prediction in various diseases^4^. AI techniques, particularly those based on machine learning and pattern recognition, can analyze large datasets to identify subtle patterns and risk factors that might be overlooked by conventional approaches. By integrating data from electronic health records, wearable devices, and patient surveys, AI models can offer a comprehensive and personalized risk assessment for developing prediabetes^14^.

The application of AI in prediabetes prediction offers several advantages, including improved accuracy, earlier intervention, and the ability to customize preventive strategies according to individual needs. This not only enhances patient outcomes but also has the potential to reduce healthcare costs by preventing the progression to type 2 diabetes and its associated complications^5^. As AI technology continues to advance, its role in predicting prediabetes represents a promising frontier in preventive medicine, providing new hope for at-risk populations and transforming the approach to managing chronic diseases.

This study focuses on the early prediction of prediabetes using an artificial intelligence model. Here, we describe how clinical data were used to develop an AI model for predicting prediabetes, using mathematical derivation. Though Previous literature has demonstrated various models for predicting prediabetes and diabetes, Our approach focuses on identifying the best model that not only accurately predicts the condition but also aligns closely with relevant clinical parameters. Additionally, our model was optimized using gradient descent with a stopping criterion to ensure robust performance.

The key contributions of this study are as follows:

- The clinical data was collected by health professionals from the patients attending Department of community medicine at GVMCH.
- The biochemical analysis for all the samples was carried out and then categorised as prediabetes and control on the basis of HbA1c.
- To initiate the AI process, the clinical data served as input features and were labelled as 0 for control subjects and 1 for individuals with prediabetes. Using these labels, the data were processed in MATLAB, yielding an output value of 0.8770. An output value greater than 0.5 was classified as prediabetes, while values below 0.5 were considered as controls.
- Pearson correlation analysis identified the strongest predictors (BMI, WC, HDL, LDL, TGL, TC, Hb, age, FBG, OGTT, VLDL and total antioxidant status) of prediabetes. These predictors were then used to develop the model, which was mathematically validated as the optimal model for prediabetes prediction.
- Interestingly TAS was observed to statistically significant (*p* < 0.0001) in this study and was a novel addition to the prediction for prediabetes. A decrease in total antioxidant status indicates increased oxidative stress, which is associated with disease progression.

This study approach will provide a clear understanding of the application of artificial intelligence in prediabetes prediction and offers an insight into the underlying clinical parameters. Early prediction of prediabetes enables timely intervention to prevent progression to diabetes. Moreover, AI models demonstrate strong capability in identifying critical features, making this network a promising tool for accurate prediabetes prediction in the clinics or hospitals.

By incorporating oxidative stress markers, this study provides the first AI model in an Indian cohort to link antioxidant status to prediabetes risk. The PNN model demonstrates excellent accuracy and interpretability, offering a clinically actionable tool for early disease detection and personalized intervention. Our approach not only achieves the state-of-the-art predictive (shown in Table 1) accuracy but also provides mechanistic insights into the role of oxidative stress in prediabetes, paving the way for targeted interventions.

**Table 1:** Comparative performance of AI models for prediabetes prediction.

Author and year	Title	Journal	Classifiers	Best accuracy	Notable features
Zueger et al.^22^	Machine learning for predicting the risk of transition from Prediabetes to Diabetes	Diabetes technology and Therapeutics	Gradient boosting model	77%	General population, transition risk
Bashar et al.^8^	A Machine learning classification model for detecting Prediabetes	Journal of data analysis and information processing	Random forest	89%	Standard clinical features
Kushwaha et al.^23^	Harnessing machine learning models for non-invasive pre-diabetes screening in children and adolescents	Computer Methods and Programs in Biomedicine	RF, GB, ETC, SVM, XGB, and DT	RF-94% GB- 94% ETC- 88% SVM- 57% XGB- 94% DT- 89%	Pediatric/adolescent focus
Kulkarni et al.^24^	Machine- learning algorithm to non- invasively detect diabetes and pre- diabetes from electrocardiogram	Digital health	XGBoost	96.8%	ECG-based, non-invasive
Tobore et al.^25^	Towards adequate prediction of prediabetes using spatiotemporal ECG and EEG feature analysis and weight-based multi-model approach	Knowledge-Based systems	Multi model: Ridge, Ada boost, support vector machine (SVM), decision tree, and neural network	95%	ECG/EEG features, multi-model
Olisah et al.^26^	Diabetes mellitus prediction and diagnosis from a data preprocessing and machine learning perspective	Computer Methods and Programs in Biomedicine	RF, SVM, and 2GDNN.	97%	Data preprocessing focus
Das et al.^29^	Prediabetes Prediction Using Response Surface Methodology and Probabilistic Neural Networks Model in an Ethnic South Indian Population	Computational Intelligence in Healthcare Informatics	ANN and PNN	95%	Salivary glucose, HbA1C, waist circumference, BMI, and LDL
Kumar et al.^30^	Comprehensive framework for thyroid disorder diagnosis: Integrating advanced feature selection, genetic algorithms, and machine learning for enhanced accuracy and other performance matrices.	Plos one	LR, RF, SVM, AB, and DT	97.21%	Hybrid framework that combines genetic algorithms with machine learning to diagnose thyroid disorders
Kumar et al.^31^	Hybrid machine learning techniques based on genetic algorithm for heart disease detection	Innovation and Emerging Technologies	LR, SVM, XGB, and GNB	94.83%	This approach combines genetic algorithms with advanced classification techniques for heart disease detection.
Li., et al.^33^	Interpretable machine learning method to predict the risk of pre-diabetes using a national-wide cross-sectional data: evidence from CHNS	BMC Public Health	XGBoost, RF, SVM, NB, ANNs, DT, and LR	93%	Age, BMI, TC, ApoB, TG, hypertension, TP, HDL-C, and WBC may serve as risk factors for pre-diabetes.
Proposed work	**Artificial Intelligence model as a tool to predict prediabetes**	–	**Proposed model: Pattern Neural Network (PNN)** **compared with SVM**,** KNN**,** and LR.**	**PNN- 98.3%**	**To include antioxidant status as predictor of prediabetes.**

*PNN* Pattern neural Network, *SVM* Support Vector Machine, *LR* Logistic Regression, *RF* Random Forest, *XGB* eXtreme Gradient boost, *DT* Decision tree, *2GDNN* Twice-growth deep neural network, *ETC* Extra Tree Classifier, *GNB* Gaussian naïve bayes, and *GB* Gradient boosting models.

## Methodology

As a pilot study, a total of 199 individuals aged 18 to 60 years were recruited. Based on HbA1c levels, participants were classified into Control (*n* = 99) and Prediabetes (*n* = 100) groups. The study received ethical approval from the Institutional Ethics Committee for Human subjects from Vellore Institute of Technology, Vellore (VIT/IECH/XIV/2023/01), Government Vellore Medical College & Hospital, Vellore (GVMCH) (VMC/III/00001/2023), and the Directorate of Public Health and Preventive Medicine, Chennai (DPH) (DPHPM/DPHSAC/2023/250). All participants were informed about the study’s objectives and procedures, and written informed consent was obtained prior to enrolment.

Peripheral blood samples (6 mL) were collected from each participant after an overnight fasting of 8 hours. Samples were drawn into clot activator tubes for biochemical analysis and EDTA vacutainers for HbA1c measurement (Guo et al., 2014)^27^. HbA1c levels were quantified using the High-Performance Liquid Chromatography (HPLC) method (Bio-Rad D-10). Fasting Blood Glucose (FBG) and Oral Glucose Tolerance Test (OGTT) levels were determined using the glucose oxidase-peroxidase (GOD-POD) enzymatic method. Lipid profiles were assessed as follows: total cholesterol by the enzymatic cholesterol oxidase-esterase peroxidase (CHOD-POD) method, triglycerides by the glycerol-3-phosphate oxidase–phenol–aminoantipyrine (GPO-TOPS) method, and HDL, LDL, and VLDL cholesterol fractions were calculated using the selective inhibition method on an automated analyzer.

In addition to standard clinical and biochemical parameters, total antioxidant scavenging potential was measured for each participant using DPPH free radical scavenging assay^28^ The serum total antioxidant status was measured using 2,2-Dipheny 1-1-picryl-hydrazyl (DPPH) free radical scavenging assay. Standard concentration of Ascorbic acid (1–10µM) is a natural antioxidant also used as positive control and 0.1 M of DPPH was used as a working solution. The serum total antioxidant status was measured by reduction in the absorbance at 517 nm using UV spectrophotometer. The serum total antioxidant status was measured by reduction in the absorbance at 517 nm using UV spectrophotometer. Then TAS was calculated using percentage scavenging potential, $\:\%\:of\:scavenging\:potential=1-\left(\frac{absorbance\:of\:sample}{absorbance\:of\:control}\right)*100\:$ and the reference range was: 20–60% for healthy individuals. This comprehensive feature set enables the model to capture a broader spectrum of risk determinants, including oxidative stress.

### Artificial intelligence models

Artificial Intelligence (AI) has revolutionized healthcare, particularly in predicting conditions such as prediabetes. Among AI techniques, pattern recognition neural networks (PNNs) have proven especially effective in this domain. These networks analyze large volumes of data to identify intricate patterns and risk factors that may not be immediately evident to healthcare providers^6^. In this study, we employed PNNs optimized via gradient descent as classifiers to develop a predictive model. The PNN model was designed with 14 input nodes, 10 hidden nodes (Tanh activation), and a single output node (sigmoid activation). The dataset was randomly split into training, validation, and testing subsets. The performance of this model was then compared with other established machine learning algorithms, as detailed below:

#### Support vector machine (SVM)

Support Vector Machine (SVM) is a supervised machine learning algorithm widely used for classification and regression tasks. It functions by identifying the optimal hyperplane that separates data points into distinct classes within a high-dimensional feature space. By utilizing kernel functions, SVM effectively manages complex, non-linear relationships, making it particularly suitable for binary classification problems^18^.

In prediabetes prediction, SVM classifies individuals based on health indicators such as age, BMI, glucose levels, and other relevant features. By analyzing these parameters, SVM aids in identifying individuals at risk of developing diabetes or prediabetes^17^. This non-invasive and efficient approach facilitates early diagnosis and intervention. Research has shown that SVM models achieve high predictive accuracy when properly tuned with appropriate kernels and hyperparameters.

#### K-Nearest neighbors (KNN)

K-Nearest Neighbors (KNN) is a simple, yet powerful supervised learning algorithm used for both classification and regression. It predicts the class of a data point based on the majority label among its ‘k’ nearest neighbors in the feature space. Being non-parametric, KNN does not assume any specific data distribution, which enhances its flexibility across diverse applications^19^. In the context of prediabetes, KNN categorizes individuals by comparing their health metrics-such as age, BMI, and glucose levels-with those of other subjects in the dataset. This comparative approach allows KNN to effectively identify individuals at risk of prediabetes^20^. Due to its simplicity and interpretability, KNN is a valuable tool for early risk assessment and timely intervention.

#### Logistic regression (LR)

Logistic Regression is a classical statistical method for binary classification that estimates the probability of an outcome based on one or more predictor variables. It models the relationship between the dependent variable (e.g., presence or absence of prediabetes or diabetes) and independent variables (e.g., glucose levels, BMI, age) using a logistic function, producing output probabilities between 0 and 1. Logistic regression is widely applied in diabetes and prediabetes prediction to analyze clinical data and identify high-risk individuals^21^. By examining key parameters such as glucose levels, BMI, and age, logistic regression provides a straightforward, interpretable, and effective approach for risk stratification. Its extensive use in medical research underscores its utility in clinical decision-making.

### Proposed model development

**Fig. 1:**
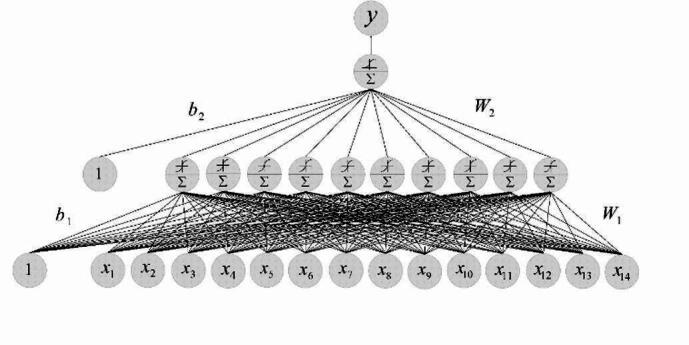
The architecture of the proposed Pattern Neural Network (PNN) model for prediabetes prediction.

A pattern neural network (PNN) is a type of artificial neural network designed to identify patterns and regularities within data^5^. Pattern Neural Networks outperform typical Artificial Neural Networks partly because they naturally incorporate cross-entropy loss during the learning phase, making them better suited for classification tasks. Cross-entropy loss is particularly useful for classification problems since it assesses how well the model’s predicted probabilities match the genuine class labels, effectively determining accurate network’s predictions^29^. In this study, we employed a PNN architecture specifically tailored for pattern recognition (Fig. 1), comprising three key layers: an input layer with *p* = 14 neurons representing the selected features, a hidden layer with **q = 10** neurons utilizing the hyperbolic tangent (Tanh) activation function to capture complex nonlinear relationships, and an output layer with a single neuron using the sigmoid activation function to produce a probability score between 0 and 1. Our model uses a Pattern Neural Network that stores each training sample as a “pattern” neuron and combined with a Bayesian decision rule. This exemplar-based approach rather than tuning abstract weight matrices gives it a unique, built-in pattern recognition mechanism for classifying prediabetes.

This configuration is well-suited for binary classification tasks, such as distinguishing between control and prediabetes cases. The dataset was randomly partitioned into training 70% (*n* = 139), validation 15% (*n* = 30), and testing 15% (30) subsets to optimize model performance and 10-fold cross-validation was performed on the training set for hyperparameter optimization. The PNN model, with its defined input, hidden, and output layers, was developed and trained to accurately predict prediabetes status.This study used mathematical representation to create a model, where the input vector was denoted as x = [x_1_ × _2_,,…x_p_]^T^, where x^i^ characterizes the i-th input feature. Then the output of the hidden layer is computed as

1 $$\:{z}^{\left(1\right)}=\:{W}^{\left(1\right)}x+{b}^{\left(1\right)}$$

also, the Tanh activation function for the hidden layer is the Tanh function, applied elementwise:

2 $$\:{A}^{\left(1\right)}=\text{t}\text{a}\text{n}\text{h}\left({z}^{\left(1\right)}\right)$$

where, $\:{\varvec{t}\varvec{a}\varvec{n}\varvec{h}}^{\left(\varvec{z}\right)}=\:\frac{{\varvec{e}}^{\varvec{z}}-{\varvec{e}}^{-\varvec{z}}}{{\varvec{e}}^{\varvec{z}}+\:{\varvec{e}}^{-\varvec{z}}}$ then the output from the hidden layer is passed to the output neuron through the weight vector *W* ^(2)^.

***y***- indicate as the output of the network is given below:

$${\varvec{z}}^{\left(2\right)}={\varvec{W}}^{\left(2\right)}\bullet\:{\varvec{A}}^{\left(1\right)}+\:{\varvec{b}}^{\left(2\right)}$$

The activation function for the output layer is the sigmoid function:

$$\:\widehat{y}=\:\varvec{\sigma\:}\left({\varvec{z}}^{\left(2\right)}\right)$$

where, $$\:\varvec{\sigma\:}\left(\varvec{z}\right)=\frac{1}{1\:+\:{\varvec{e}}^{-\varvec{z}}}$$

Then through backpropagation, the networks trained the appropriate weights W^(1)^, W^(2)^ and biases b^(1)^, b^(2)^. During the training phase of the dataset, binary cross-entropy loss was used to minimize the loss of function, which is given below:

$$\:\varvec{L}=\:-\frac{1}{\varvec{m}}{\sum\:}_{\varvec{i}=1}^{\varvec{m}}[{\varvec{y}}^{\left(\varvec{i}\right)}\mathbf{log}{\widehat{\varvec{y}}}^{\left(\varvec{i}\right)}+(1-{\varvec{y}}^{\left(\varvec{i}\right)}\mathbf{log}(1-{\widehat{\varvec{y}}}^{\left(\varvec{i}\right)})]$$

## The proposed framework

### Data

The samples were processed at the BMGRL, Vellore Institute of Technology (VIT) and the parameters were used to build a model. In this study, 14 parameters were considered to predict prediabetes clinically, age, gender, TAS, HbA1c, FBG, OGTT, TGL, HDL, LDL, VLDL, TC WC, Hb, and BMI. These parameters were considered as an input feature (p) and the hidden nodes were taken by random selection (q = 10).

### Data preprocessing

**Fig. 2:**
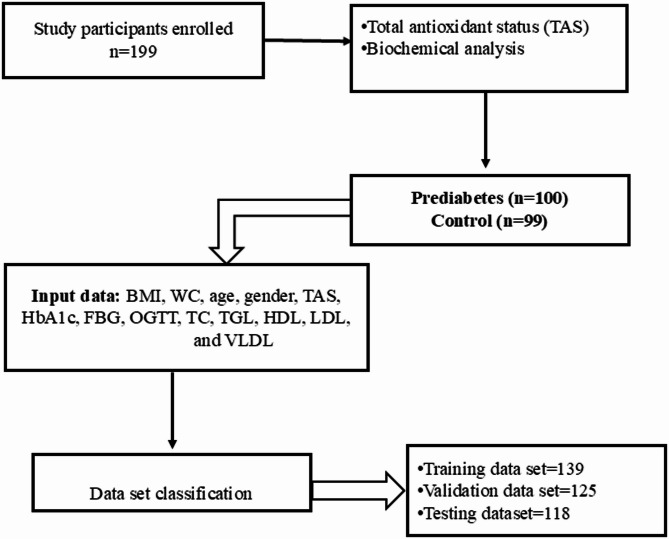
Details of samples used for data analysis and classification.

Figure 2 illustrates the overall analytical pipeline and preprocessing steps implemented in this study. The model architecture included a hidden layer with 10 nodes (q = 10) to facilitate the learning of complex patterns.Data were randomly partitioned into three distinct sets: training, validation, and testing, with 139, 125, and 118 samples, respectively. All clinical and demographic data were carefully preprocessed, including the handling of missing values, outlier detection, and feature normalization. The training set was used to develop the Pattern Neural Network (Patternet) model, employing a cross-entropy loss algorithm for optimal prediction of prediabetes.

### Model optimization

Gradient descent with stopping criteria was used as model optimization to minimize the loss function in neural networks by repetitively adjusting the network’s weights and biases, whereas the input dataset includes a loss function to minimize *f*(*W*), gradient of the function ∇*f*(*W*), initial weights *w*_0,_ learning rate η, number of iterations mixites and validation data was (X_val_, Y_val_). For a binary classification task, the network’s objective is to minimize the binary cross-entropy loss function, defined in Eq. (5).

Where, y_true_ is the true label (0 or 1) and y_pred_ is the predicted probability output from the network.

The gradient descent algorithms update the weights $\:{W}^{\left(1\right)}$, $\:{W}^{\left(2\right)}$and biases $\:{b}^{\left(1\right)}$, $\:{b}^{\left(2\right)}$by computing the gradient of the loss function with respect to the parameters listed below:

$$\:{W}_{2}={W}_{2}-\eta\nabla\:W$$

$$\:{b}_{2}={b}_{2}-\eta\nabla\:{b}_{2}$$

$$\:{W}_{1}={W}_{1}-\eta\nabla\:{W}_{1}$$

$$\:{b}_{1}=\:{b}_{1}-\eta\nabla\:{b}_{1}$$

### Model validation

The model was evaluated using a confusion matrix during training (139) dataset against the validation set. In this study, a confusion matrix was used to validate the training, validation, and test dataset (shown in Fig. 5). The validation data performance of the model was evaluated using four different matrices:

- Precision: This is the proportion of patients with prediabetes, the positive instance, who are correctly identified as being prediabetic out of all the prediabetic patients and is comparable to positive predictive value in epidemiology and computed as the ratio of true positive (TP) to the sum of TP and false positive (FP).

$$\:Precision=\:\frac{TP}{TP+FP}$$

(b) Recall: This is the proportion of patients with prediabetes, the positive instances, who are correctly identified as being prediabetic and it is computed as comparable to sensitivity in epidemiology$\:Recall\:or\:senstivity=\:\frac{TP}{FN+TP}$.
(c) Accuracy: Ratio of the total number of predictions, and it is expressed negatives.

$$\:Accuracy=\:\frac{TP+TN}{TP+FP+FN+TN}$$

(d) F1 score: This is the weighted average of precision and recall. As a result, this score considers both false positives and false negatives.

$$\:F1-score=\:2\times\left[\frac{\left(Recall\times\:Precision\right)}{Recall+Precision}\right]$$

Here, TP represents true positive, FP- False positive, TN- True negative, and FN- False negative. ROC curve is used to evaluate the performance of a binary diagnostic classification method. Figure 6 shows the performance of the model by plotting the true positive rate (TPR) on the y-axis and the false positive rate (FPR) on the x-axis. The area under the ROC curve is known as AUC which implies the degree of separability that provides how much a model is capable of distinguishing between individuals. A higher value of AUC shows a better prediction model.

### Data analysis

Mean and standard deviation for all measured clinical and biochemical characteristics were calculated using the student’s t-test (GraphPad). To visually summarize the data, box plots were employed, providing an effective graphical representation of the distribution and skewness of numerical variables^15^. Each box plot displays the five-number summary: minimum, lower quartile (Q1), median, upper quartile (Q3), and maximum. Figure 7 presents the box plot visualization of the key biochemical parameters.

Additionally, the Pearson correlation coefficient was used to assess the linear relationship between pairs of variables^16^. Pearson correlation analysis was performed on biochemical, clinical, and percentage scavenging potential data among individuals with prediabetes. The results revealed moderate to strong correlations among several parameters, as detailed in Table 5.

## Results

### Study participants

As depicted in Fig. 2, data from 199 eligible participants were analyzed in this study. The cohort included 99 individuals in the control group (44 males, 55 females) and 100 individuals with prediabetes (39 males, 61 females), and all aged between 18 and 60 years. Fourteen clinical and biochemical characteristics were assessed as potential predictors of prediabetes (Table 2), as clearly established risk factors in recent literature.

Descriptive statistics, including mean and standard deviation, were calculated for all variables using GraphPad Student’s t-test calculator, as presented in Table 2. Among the 14 evaluated characteristics, age, BMI, waist circumference (WC), percentage scavenging potential, HbA1c, and oral glucose tolerance test (OGTT) values demonstrated statistically significant differences between groups (*p* < 0.05). These findings are consistent with previous studies that underscore the importance of these parameters in identifying individuals at increased risk for prediabetes and highlight their value in predictive modeling and early intervention strategies.

**Table 2:** Demographic, anthropometric and biochemical parameters.

Characteristic	Control	Prediabetes	*p*-value
Male	44	39	
Female	56	61	
Age(years)	38.3 ± 9.45	41.4 ± 8.71	0.001*
Body Mass Index (BMI) (Kg/m^2^)			
Male	25.577 ± 4.195	28.969 ± 4.939	0.0011*
Female	27.159 ± 5.321	29.838 ± 5.49	0.0086*
Waist circumference (WC) (cm)			
Male	82.386 ± 13.158	98.903 ± 16.603	0.0001*
Female	85.161 ± 13.952	98.807 ± 14.458	0.0001*
Total antioxidant status (TAS) %	36.980 ± 13.362	14.257 ± 8.360	0.0001*
Biochemical analysis			
Fasting blood glucose(mmol/L)	85.97 ± 16.50	89.21 ± 20.66	0.22
Oral glucose tolerance test(mmol/L)	108.60 ± 23.37	120.83 ± 28.3	0.0011*
HbA1c %	5.31 ± 0.24	5.96 ± 0.25	0.0001*
Total serum cholesterol (mg/dL)	175.8 ± 34.8	185.9 ± 49.1	0.09
Serum triglycerides (mg/dL)	133.4 ± 72.7	147.9 ± 83.5	0.1
Serum HDL (mg/dL)	44.20 ± 10.4	43.72 ± 13.8	0.7
Serum LDL (mg/dL)	105.03 ± 29.2	112.3 ± 36.02	0.1
Serum VLDL (mg/dL)	26.32 ± 13.9	29.4 ± 16.3	0.1
Hemoglobin (gm/dL)	14.03 ± 0.828	12.49 ± 2.107	0.07

Student t test.

p value < 0.05, significant.

### Statistical analysis for biochemical parameters

#### Boxplot analysis

**Fig. 3:**
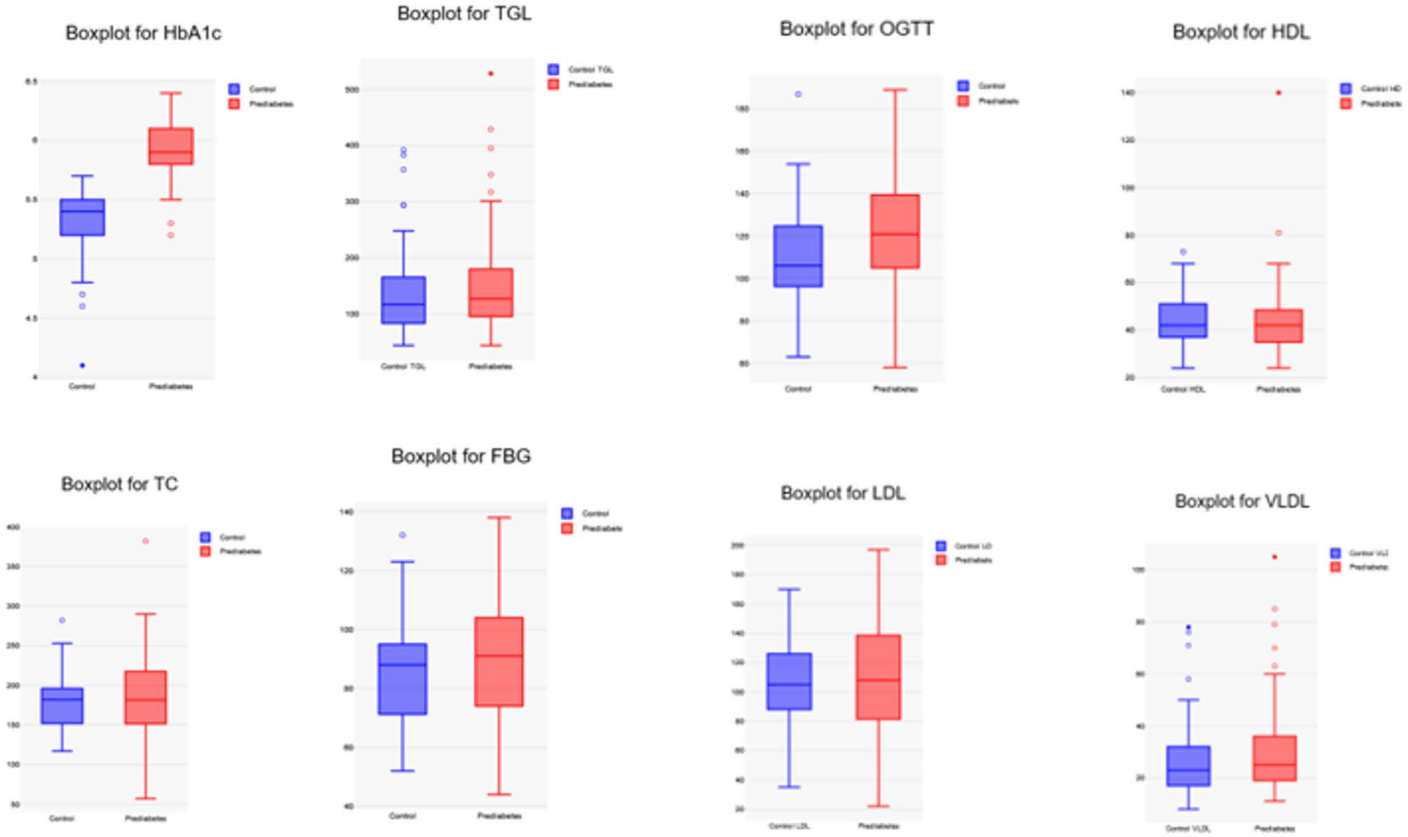
Box plot visualization of Control and Prediabetes for biochemical parameters.

Figure 3 presents box plots illustrating the distribution of key biochemical parameters, including HbA1c, triglycerides (TGL), total cholesterol (TC), HDL, LDL, fasting blood glucose (FBG), oral glucose tolerance test (OGTT), and VLDL. Notably, the median HbA1c value for the prediabetes group lies outside the interquartile range of the control group, highlighting a clear distinction between these two populations. Greater interquartile range (IQR) lengths observed for FBG, OGTT, and LDL indicate increased data dispersion and variability in these markers between control and prediabetes groups.

The analysis also revealed positive skewness in several parameters, as detailed in Table 2, suggesting an asymmetric distribution. Among the biochemical parameters, HDL levels in the prediabetes group exhibited a distribution close to normal or slight positive skewness, as shown in Tables 3 and 4. These visual and statistical insights underscore the significant biochemical differences between control and prediabetes groups, supporting their utility as discriminative markers for early risk assessment.

**Table 3:** The biochemical parameters for control individuals using box plot analysis.

S.no	Box-plot attribute	Outcome	Min	1 st quartile	Median	3rd quartile	Max	Mean	Skewness	Outliers
**1.**	**HbA1c %**	Control	4.1	5.2	5.4	5.5	5.7	5.3162	−1.651	4.1,4.6,4.7
**2.**	**TGL** (mg/dL)	Control	44	83.5	117	165.5	392	133.44	1.4714	392,357,294, 294,383
**3.**	**TC** (mg/dL)	Control	117	152	182	196	282	175.8081	0.363	282
**4.**	**FBG** (mmol/L)	Control	52	71.25	88	95	132	85.9697	0.069934	132
**5.**	**OGTT** (mmol/L)	Control	63	96.25	106	124.75	187	108.596	0.2809	187
**6.**	**HDL** (mg/dL)	Control	24	37	42	51	73	44.202	0.4076	73
**7.**	**LDL** (mg/dL)	Control	35	88	105	126	170	105.03	−0.1208	–
**8.**	**VLDL** (mg/dL)	Control	8	17	23	32	78	26.3293	1.5232	78,71,58,76

**Table 4:** The biochemical parameters for prediabetes individuals using box plot analysis.

S.no	Box-plot attribute	Outcome	Min	1 st quartile	Median	3rd quartile	Max	Mean	Skewness	Outliers
**1.**	**HbA1c %**	Prediabetes	5.2	5.8	5.9	6.1	6.4	5.964	−0.00770	5.2,5.3
**2.**	**TGL** (mg/dL)	Prediabetes	44	95.5	127	180	528	147.98	1.9207	317,395,528, 348,429
**3.**	**TC** (mg/dL)	Prediabetes	57	151.5	181.5	217.5	382	185.91	0.5673	382
**4.**	**FBG** (mmol/L)	Prediabetes	44	74	91	104	138	89.27	−0.1468	–
**5.**	**OGTT** (mmol/L)	Prediabetes	58	105	121	139.5	189	120.83	−0.00969	–
**6.**	**HDL** (mg/dL)	Prediabetes	24	35	42	48.5	140	43.72	3.6909	81, 140
**7.**	**LDL** (mg/dL)	Prediabetes	22	81.5	108	138.5	197	112.35	0.2624	–
**8.**	**VLDL** (mg/dL)	Prediabetes	11	19	25	36	105	29.94	1.9742	63,79,105,70, 85

#### Correlation analysis

Pearson correlation (r) coefficient was carried out, observed positive correlation between age and waist circumference was 0.2028 (*P* = 0.001 95% CI). Whereas increased BMI correlates strongly with waist circumference (0.4226) and BMI with FBG (0.2063). Then waist circumference shows a weak positive correlation with FBG, 0.2335 (*P* = 0.001 95% CI) (Table 5).

**Table 5:** Correlation matrix for biochemical, demographic, and clinical parameters of prediabetes.

Parameters	TAS	Age	BMI	Waist circumference
Mean Age	−0.0101			
BMI (kg/m^2^)	−0.0566	−0.006		
Waist circumference (cm)	−0.1718	0.2028*	0.4226*	
HbA1c (%)	0.0335	0.1501	−0.0228	0.073
FBG (mmol/L)	−0.1744	0.186	0.2063*	0.2335*
OGTT (mmol/L)	0.0876	0.1251	0.1402	0.0048
TGL (mg/dL)	0.0143	−0.0144	−0.1484	−0.2135
HDL (mg/dL)	0.1475	0.0508	−0.2575	−0.1752
LDL (mg/dL)	−0.0288	0.1176	−0.0439	0.0677
VLDL (mg/dL)	0.0388	−0.0052	−0.1627	−0.2218
Cholesterol (mg/dL)	0.0651	0.0793	−0.1077	−0.1

* *p* < 0.05.

### Training and predictive performance

**Fig. 4:**
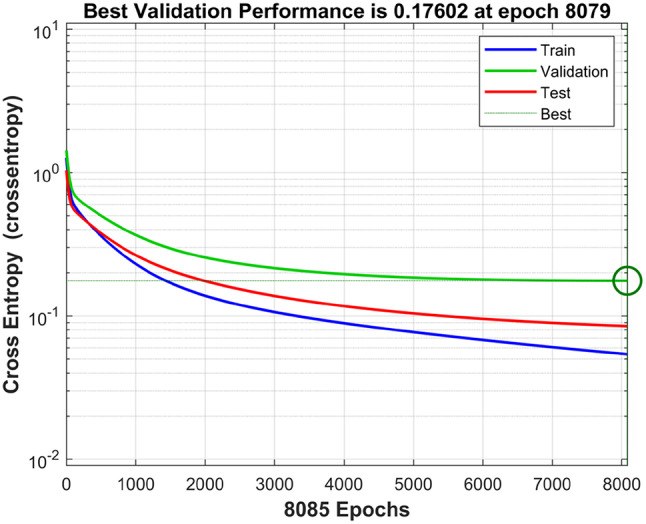
Performance curves with best validation performance.

**Fig. 5:**
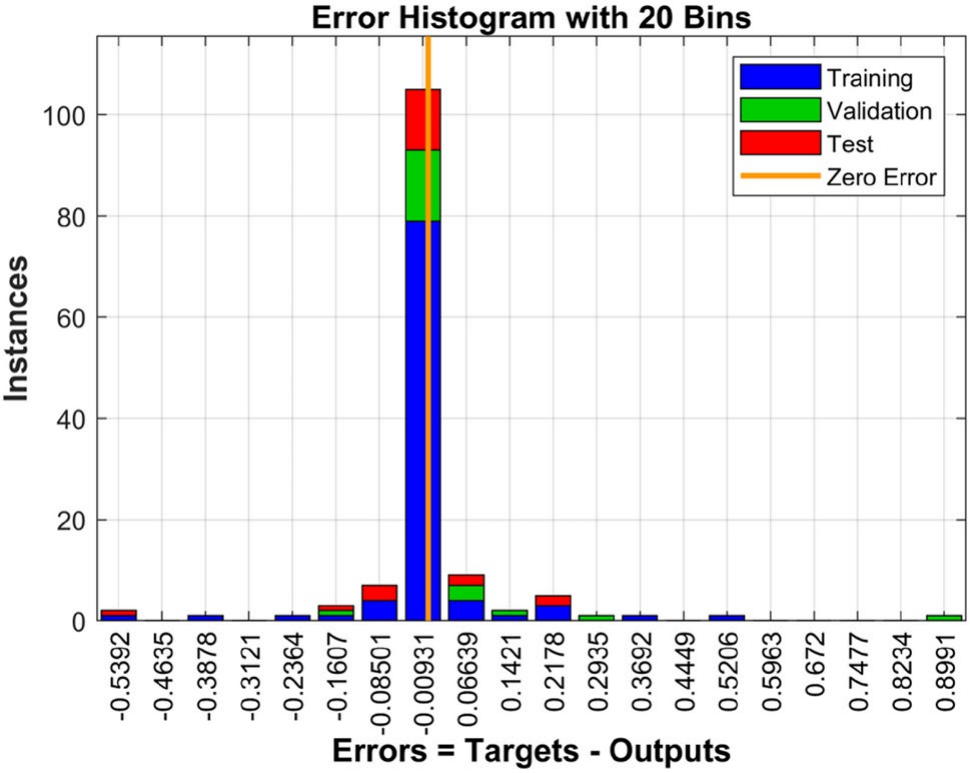
Error histogram of training, validation and testing dataset.

To develop and evaluate the predictive model, the dataset was randomly partitioned into training, validation, and testing subsets. Of the total data, 139 samples were allocated for model training (Fig. 2). Individuals were labeled as control (0) or prediabetes (1) based on clinical criteria. The Pattern Neural Network (PNN) model demonstrated efficient and stable training, achieving optimal validation performance with a loss of 0.17602 at epoch 8,079 (Fig. 4). The error histogram (Fig. 5) was centered near zero (− 0.00931), indicating minimal error during training. Errors were quantified as the difference between target and output values.

**Fig. 6:**
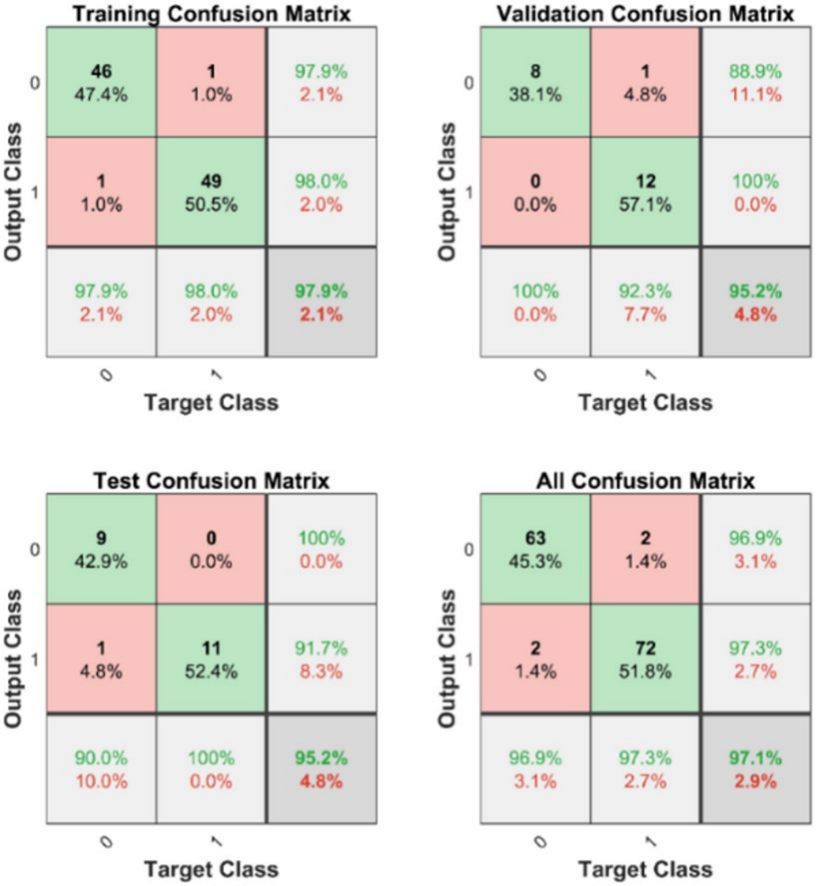
Confusion matrices for the predictive performance of the model in training, validation, test set, and all confusion matrices.

The model’s predictive performance is summarized in Fig. 6, which displays confusion matrices for the training, validation, testing, and combined datasets. The PNN algorithm effectively distinguished between control and prediabetes classes, as evidenced by the strong diagonal aggregation in the confusion matrices. The classification accuracy exceeded 97.9% in the training set, 95.2% in the validation set, and 95.2% in the testing set, highlighting the model’s robust generalizability and discriminative power.

Input features included BMI, waist circumference, age, gender, percentage scavenging potential, HbA1c, fasting blood glucose (FBG), oral glucose tolerance test (OGTT), triglycerides (TG), HDL, LDL, total cholesterol (TC), hemoglobin (Hb), and VLDL. This comprehensive set of clinical and biochemical predictors enabled the model to capture complex patterns associated with prediabetes risk, supporting its utility as a reliable screening tool.

This study used mathematical representation to create a model, where the input vector was denoted as x = [x_1_ × _2_,…x_p_]^T^, where x^i^ characterizes the i-th input feature. Our final model equation was given in (2.2 proposed model development) as Eq. 4. Here we have mentioned the overall derivation of our proposed model.

$$\:\varvec{y}\left(\varvec{x}\right)=\varvec{\sigma\:}(\varvec{W}{2}^{\varvec{T}}\mathbf{tanh}\left(\varvec{W}{1}^{\varvec{T}}\mathbf{x}+{\varvec{b}}_{1}\right)+{\varvec{b}}_{2})\:\:$$

where,

$$\:x=\left[\begin{array}{c}{x}_{1}\\\:{x}_{2}\:\\\:{\begin{array}{c}\vdots \\\:x\end{array}}_{14}\end{array}\right],$$

$$\:\varvec{W}1=\left[\begin{array}{c}-0.21\\\:0.92\\\:\begin{array}{c}0.36\\\:-0.45\\\:\begin{array}{c}0.38\\\:0.71\\\:\begin{array}{c}-0.11\\\:-0.34\\\:\begin{array}{c}-0.51\\\:0.15\end{array}\end{array}\end{array}\end{array}\end{array}\begin{array}{c}0.11\\\:-0.31\\\:\begin{array}{c}-0.03\\\:0.24\\\:\begin{array}{c}0.05\\\:-0.56\\\:\begin{array}{c}-0.38\\\:-0.47\\\:\begin{array}{c}0.18\\\:0.21\end{array}\end{array}\end{array}\end{array}\end{array}\begin{array}{c}\begin{array}{c}\begin{array}{c}\begin{array}{c}\begin{array}{c}-0.48\\\:-0.85\end{array}\\\:-0.62\end{array}\\\:-1.21\\\:1.33\end{array}\\\:0.41\\\:-0.01\end{array}\\\:-0.17\\\:\begin{array}{c}-0.18\\\:-1.04\end{array}\end{array}\begin{array}{c}\begin{array}{c}\begin{array}{c}\begin{array}{c}\begin{array}{c}0.58\\\:0.57\end{array}\\\:1.34\\\:0.11\end{array}\\\:-0.64\\\:0.14\end{array}\\\:0.34\\\:0.35\end{array}\\\:-0.52\\\:2.57\end{array}\begin{array}{c}-0.53\\\:0.41\\\:\begin{array}{c}-0.04\\\:-0.06\\\:\begin{array}{c}-0.01\\\:-0.34\\\:\begin{array}{c}-0.36\\\:-0.21\\\:\begin{array}{c}-0.56\\\:-0.33\end{array}\end{array}\end{array}\end{array}\end{array}\begin{array}{c}\begin{array}{c}\begin{array}{c}\begin{array}{c}\begin{array}{c}-0.54\\\:0.42\end{array}\\\:0.46\\\:0.33\end{array}\\\:-0.11\\\:0.62\end{array}\\\:0.81\\\:-0.42\end{array}\\\:0.18\\\:-0.25\end{array}\begin{array}{c}\begin{array}{c}\begin{array}{c}\begin{array}{c}\begin{array}{c}-0.61\\\:0.21\end{array}\\\:-0.19\\\:0.02\end{array}\\\:0.41\\\:0.17\end{array}\\\:0.21\\\:0.61\end{array}\\\:0.62\\\:0.13\end{array}\begin{array}{c}-0.42\\\:-0.74\\\:\begin{array}{c}0.57\\\:-0.57\\\:\begin{array}{c}-0.22\\\:-0.48\\\:\begin{array}{c}0.91\\\:0.31\\\:\begin{array}{c}0.13\\\:-0.10\end{array}\end{array}\end{array}\end{array}\end{array}\begin{array}{c}-0.52\\\:0.36\\\:\begin{array}{c}-0.33\\\:-0.66\\\:\begin{array}{c}0.16\\\:-0.64\\\:\begin{array}{c}0.61\\\:0.51\\\:\begin{array}{c}0.24\\\:-0.52\end{array}\end{array}\end{array}\end{array}\end{array}\begin{array}{c}\begin{array}{c}\begin{array}{c}\begin{array}{c}\begin{array}{c}0.15\\\:-0.003\end{array}\\\:0.82\\\:0.36\end{array}\\\:-0.01\\\:-0.05\end{array}\\\:-0.11\\\:0.62\end{array}\\\:-0.67\\\:-0.35\end{array}\begin{array}{c}-0.41\\\:-0.26\\\:\begin{array}{c}0.51\\\:0.38\\\:\begin{array}{c}-0.61\\\:-0.15\\\:\begin{array}{c}0.06\\\:-0.11\\\:\begin{array}{c}0.21\\\:0.05\end{array}\end{array}\end{array}\end{array}\end{array}\begin{array}{c}-0.25\\\:-0.11\\\:\begin{array}{c}-0.58\\\:0.62\\\:\begin{array}{c}-0.46\\\:-0.59\\\:\begin{array}{c}-0.21\\\:-0.54\\\:\begin{array}{c}-0.004\\\:-0.15\end{array}\end{array}\end{array}\end{array}\end{array}\begin{array}{c}0.55\\\:0.67\\\:\begin{array}{c}-0.43\\\:0.34\\\:\begin{array}{c}-0.51\\\:-0.15\\\:\begin{array}{c}0.05\\\:0.55\\\:\begin{array}{c}-0.23\\\:0.55\end{array}\end{array}\end{array}\end{array}\end{array}\begin{array}{c}\begin{array}{c}\begin{array}{c}\begin{array}{c}\begin{array}{c}-0.27\\\:0.51\end{array}\\\:0.79\\\:0.03\end{array}\\\:-0.05\\\:0.26\end{array}\\\:-0.61\\\:-0.36\end{array}\\\:0.78\\\:0.05\end{array}\right]$$

$$\:\varvec{b}1=[{\begin{array}{ccc}1.71&\:-0.99&\:\begin{array}{ccc}0.64&\:0.43&\:\begin{array}{ccc}0.22&\:0.11&\:\begin{array}{ccc}-0.55&\:-0.96&\:\begin{array}{cc}-1.44&\:-1.81\end{array}\end{array}\end{array}\end{array}\end{array}]}^{T}$$

$$\:\varvec{W}2=[{\begin{array}{ccc}-1.05&\:1.10&\:\begin{array}{ccc}2.03&\:1.07&\:\begin{array}{ccc}-1.60&\:0.19&\:\begin{array}{ccc}0.28&\:1.11&\:\begin{array}{cc}-1.11&\:3.07\end{array}\end{array}\end{array}\end{array}\end{array}]}^{T}$$

$$\:\varvec{b}2=0.22861$$

For instance,

$$\:X=\left[\begin{array}{ccc}32&\:0&\:\begin{array}{ccc}38&\:5.3&\:\begin{array}{ccc}103&\:105&\:\begin{array}{ccc}50&\:52&\:\begin{array}{ccc}103&\:10&\:\begin{array}{ccc}165&\:12.3&\:\begin{array}{cc}84&\:25.1\end{array}\end{array}\end{array}\end{array}\end{array}\end{array}\end{array}\right]{\:}^{T}$$

$$\:\widehat{\varvec{y}}\approx\:0.877$$

In our model validation, the output value is greater than 0.5, which shows the individuals are at a risk of developing diabetes in future. This model shows best validation performance.

### Model validation

**Fig. 7:**
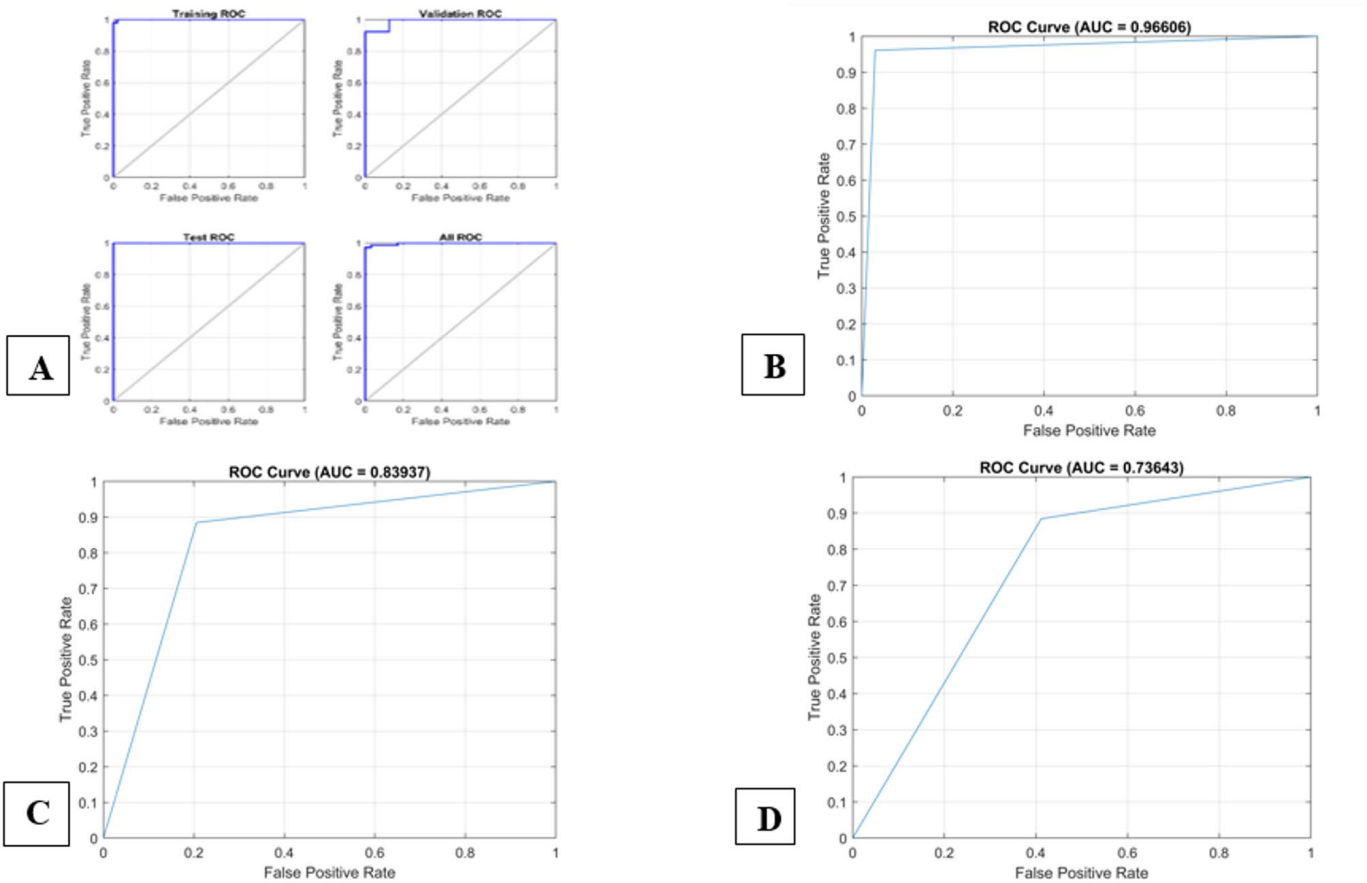
ROC curve comparison of classifiers of dataset.

The proposed framework was compared with SVM, KNN, and LR to prove the accuracy of the proposed model. Figure 7A shows, higher value of AUC among training, validation, and testing datasets. These confusion matrices show performance of the proposed model which was evaluated using multiple accuracy parameters and represented in the Table 6, including overall accuracy (0.98333), precision (1), recall(0.96154), F1-score (0.98039), and area under the Receiver Operating Characteristic (ROC) curve (AUC-ROC). 7(B) shows the SVM classifier ROC curve, AUC = 0.96606, 7(C) shows the KNN classifier ROC cure, AUC = 0.83937, and 7(D) represents the logistic regression ROC curve, AUC = 0.73643.

**Table 6:** Performance metrics of classifiers for prediabetes Prediction.

Classifiers	Accuracy	Precision	Re-call	F1-score	AUC
PNN	0.98333	1	0.96154	0.98039	0.9808
SVM	0.96607	0.96154	0.5814	0.41667	0.96606
KNN	0.83333	0.7664	0.38333	0.51111	0.83937
LR	0.71667	0.62162	0.38333	0.47423	0.73643

### Limitations and future work

The study includes numerous limitations: (1) the single-center approach might limit generalizability; (2) the moderate sample size (*n* = 199) requires validation in larger cohorts; and (3) the cross-sectional methodology hinders the investigation of temporal connections. Future study should include multicentred validation, long-term follow-up, and integration with additional biomarkers.

## Discussion

This is the first study to integrate total antioxidant status of the prediabetes patients into an AI-driven prediction model in an Indian cohort. The inclusion of oxidative stress markers provides a mechanistic link between metabolic dysregulation and disease risk, offering a new dimension for risk stratification and personalized intervention.

A study conducted in Romania identified triglycerides, HDL, waist circumference (WC), glucose, and gender as significant risk factors for developing a neural network-based predictive model for prediabetes^7^. Building on this, our study incorporated a comprehensive set of variables including age, BMI, WC, biochemical parameters (triglycerides, total cholesterol, LDL, HDL, VLDL, HbA1c, fasting blood glucose, oral glucose tolerance test, and hemoglobin), as well as percentage scavenging potential to evaluate oxidative stress levels in individuals.

Our findings revealed significantly low antioxidant levels in prediabetic individuals (14.257 ± 8.360) compared to controls (36.980 ± 13.362), indicating elevated oxidative stress, which is known to contribute to the progression of metabolic complications. Notably, BMI and waist circumference emerged as the strongest anthropometric predictors of prediabetes, particularly in the 17–19-year age group. ROC analysis further confirmed BMI as a robust predictor of prediabetes risk^12^. Similarly, a study on the Chinese Han population reported a significant positive correlation between BMI, waist circumference, and prediabetes risk^13^. Similarly, In our study, Pearson correlation analysis demonstrated a strong association between BMI and waist circumference among prediabetic subjects, underscoring their value as key predictive markers.

Previous studies have reported varying predictive accuracies using different AI models: a Romanian population study achieved 80–96% accuracy for model validation^7^, and a US-based study using Random Forest models reported approximately 89% accuracy^8^. ANN and SVM have also been applied, yielded accuracies of 65.6% and 69.9%, with AUC values of 0.706 and 0.742, respectively^9^. Other machine learning algorithms, including logistic regression, naïve Bayes, Random Forest, XGBoost, and extremely randomized trees, have demonstrated accuracies ranging from 66% to 82% for prediabetes prediction^10^. In a Chinese cohort, the GA_XGBT model showed high precision (0.929), recall (0.951), and an F1-score of 0.94^11^. Another study by Kumar et al. (2025) reported 88% accuracy and 100% precision in detecting arrhythmia, highlighting its potential in clinical applications implies the impact of oxidative stress^32^.

In this study, PNN model was utilized for predicting prediabetes in individuals aged 18 to 60 years. This model achieved superior performance, with an accuracy of 98%, precision of 1.0, recall of 0.9615, and an F1-score of 0.9804, indicating its strong predictive capability. To regularize the model, we monitor validation performance and stop training if the cross-entropy loss fails to improve or worsens over six consecutive iterations, thereby avoiding convergence to suboptimal local minima (Fig. 4). This will be predictive tool for early diagnosis and management of prediabetes. It is also less time consuming and less expensive. While the model demonstrates excellent performance, further validation in larger, multi-centre cohorts is warranted to confirm generalizability. Future work will focus on external validation, integration with electronic health records, and prospective evaluation in clinical settings.

## Conclusion

In this study, BMI, waist circumference (WC), age, percentage scavenging potential, HbA1c, fasting blood glucose (FBG), and oral glucose tolerance test (OGTT) emerged as key risk factors associated with the progression from prediabetes to diabetes. Our primary objective was to identify the most significant predictors among 14 clinical and biochemical characteristics and mathematically represent them using a Pattern Neural Network (PNN) model.

This study shows the importance of using oxidative stress markers into an AI model, achieving superior accuracy and clinical interpretability. The proposed PNN model offers a robust, actionable tool for early identification and intervention, with the potential to transform preventive strategies in high-risk populations and reduce risk for progressing to diabetes.

The performance of the PNN model was rigorously evaluated using confusion matrices and key metrics including precision, accuracy, recall, and F1-score. The PNN demonstrated superior validation performance across all these indicators, establishing it as the most effective model for predicting prediabetes in our dataset. While the SVM model exhibited high precision and overall accuracy, it showed comparatively lower recall and F1-score values. Similarly, KNN and LR models had reduced recall and AUC values, suggesting they may require further optimization to achieve comparable predictive power.

A strong correlation between BMI and waist circumference further underscores their critical role as a major anthropometric risk factors for prediabetes development. Given its outstanding validation accuracy and balanced performance metrics, the PNN model holds significant promise as a reliable tool for early prediabetes prediction and risk stratification.

## Data Availability

The datasets generated and/or analysed during the current study are not publicly available due confidentiality of the patient’s information but are available from the corresponding author on reasonable request.
